# Toxinotyping and Sequencing of *Clostridium difficile* Isolates from Patients in a Tertiary Care Hospital of Northern India

**DOI:** 10.3389/fmed.2017.00033

**Published:** 2017-03-28

**Authors:** Meenakshi Singh, Chetana Vaishnavi, Safrun Mahmood, Rakesh Kochhar

**Affiliations:** ^1^Department of Gastroenterology, Postgraduate Institute of Medical Education and Research, Chandigarh, India; ^2^Department of Experimental Medicine and Biotechnology, Postgraduate Institute of Medical Education and Research, Chandigarh, India

**Keywords:** *Clostridium difficile*, sequencing, toxinotyping, *tcd*A, *tcd*B

## Abstract

**Background:**

*Clostridium difficile* is an important cause of infectious colitis among hospitalized patients across the globe. The pathogenic potential of *C. difficile* in producing significant morbidity and mortality is mainly due to production of toxins A and B. The outbreaks of *C*. *difficile* infection (CDI) are due to changes in the genetic sequences of the organism. There is hardly any molecular study reported on the prevalent types of *C. difficile* strains in India. Toxinotyping and sequencing of locally circulating *C*. *difficile* isolates from patients presenting to our tertiary care center of North India were done.

**Materials and methods:**

*C. difficile* strains (*n* = 174) isolated from 1,110 fecal samples from patients with suspected CDI were subjected to toxinotyping and partial sequencing of *tcd*A and *tcd*B genes. Comparison of nucleotide sequences with reference *C. difficile* 630 strain using BLAST was made and translated into corresponding amino acid sequences by ExPASy.

**Results and discussion:**

Of 174 *C. difficile* isolates, 121 were toxigenic, belonging to toxinotype 0 (*n* = 76) and VIII (*n* = 45). Partial sequencing of toxin genes using bioinformatics approaches revealed changes in toxin A sequences of five (50%) *C. difficile* isolates, but the translated nucleotide sequences showed substitution in only three of them. No variation was seen in the toxin B nucleotide sequences. Interstrain variations were found in the clinical *C. difficile* isolates in our region.

**Conclusion:**

PCR amplified toxigenic genes followed by sequencing can help to identify genetic changes and pathogenicity of varied collection of *C. difficile* isolates.

## Introduction

*Clostridium difficile*, an anaerobic spore-bearing organism, is an important cause of infectious colitis among in-patients across the globe and particularly so in North America and Europe (1). The increase in the morbidity and mortality due to *C. difficile* is worrisome. Clinically significant strains of *C. difficile* are capable of producing two major toxins: toxin A (*TcdA*) and toxin B (*TcdB*), which are encoded by *tcd*A and *tcd*B genes, respectively, present on the 19.6 kb pathogenicity locus (PaLoC) of *C. difficile* chromosome (2, 3). From 2003 onward, several outbreaks have been reported due to NAP1/BI/027, a high-level toxin-producing *C. difficile* strain isolated from North America (3), Europe (4), and Japan (5). NAP1/BI/027 is more prolific in sporulation than its non-hypervirulent counterpart and produces more amount of toxins than the historical strain (4). The *C*. *difficile* infection (CDI) outbreaks are due to changes in the genetic sequences called the “genetic switch” (6). Comparison of whole genome sequencing of historical strain with the hypervirulent strain showed that *C*. *difficile* had acquired genes that enabled it to survive better in the environment (7). This hypervirulent strain is also linked with a more serious course of infection, higher relapse rate, and complications leading to higher mortality (8).

Apart from the NAP1/BI/027, several other *C. difficile* strains have been reported from various countries (1). Also, strains that produce only toxin B but not toxin A have appeared in Asia (9) and Latin America (10). The occurrence of different *C. difficile* strains circulating globally has led to the advancement of methods for a better insight into the pathogenicity of various strains in the etiology of nosocomial outbreaks and to provide a better follow-up of the epidemiology of the disease (11).

There is hardly any molecular study reported on the prevalent types of *C. difficile* strains in India (9). In the wake of several global outbreaks, the locally circulating strain types of *C. difficile* from patients presenting to a tertiary care center of North India were investigated.

## Materials and Methods

### Patient Population

The study protocol was approved by the Institute Ethics Committee and was carried out in accordance with the recommendations of ICMR Ethical Guidelines for Biomedical Research in India, New Delhi, with written informed consent from all subjects in accordance with the Declaration of Helsinki. A total of 1,110 (M:F = 1.8:1; age range >2–95 years) consecutive hospitalized patients who developed diarrhea after ≥72 h of admission in various wards and suspected of CDI by the clinicians were enrolled for the investigation (12). Patients with incomplete data, pregnant women, and children <2 years were excluded (12). Apart from Chandigarh, our tertiary care referral hospital accommodates patients from various regions of North India inclusive of Jammu and Kashmir, Punjab, Haryana, Himachal Pradesh, Uttar Pradesh, and Rajasthan as reported earlier (12). The patients were clinically evaluated for CDI symptoms inclusive of fever and pain abdomen by the clinicians. CDI was diagnosed/suspected by the clinicians based on clinical signs and symptoms (diarrhea/fever/abdominal pain/antibiotic exposure) and/or endoscopic evaluation.

A total of 174 *C. difficile* isolates were obtained after culture of single fecal sample per patient from 1,110 patients. After phenotypic and genotypic confirmation (12) and amplification of *tcd*A and *tcd*B genes (13), the *C. difficile* isolates were subjected to toxinotyping and partial sequencing.

### Toxinotyping

Toxigenic *C. difficile* isolates were identified for changes in PaLoc region by toxinotyping method (14). The entire *tcd*A and *tcd*B genes were amplified using six different overlapping PCRs (Table 1). In brief, isolation of DNA was carried out by the phenol–chloroform technique, and PCR amplification was carried out in a Mastercycler (Eppendorf, Germany). PCRs were done in 50 μl volume containing DNA (300 ng), each paired primer (15 pmol), a concentration of each deoxynucleoside triphosphate (200 μM), and Taq polymerase (2.5 U). Two-step PCR programs used included initial denaturation at 93°C for 3 min and annealing and extension for 8 min at 56°C (fragments A1 and A2) or at 47°C (fragments A3, B1, B2, B3), followed by denaturation at 93°C for 4 s. Agarose (1.5%) was used to visualize the size differences in the amplified fragments, which were further subjected to digestion with different restriction enzymes to identify polymorphism.

**Table 1:** **Primers used for identification of toxigenic genes (fragments)**.

Target gene	Primers	Sequence 5′➔3′	Annealing temperature (°C)	Amplicon size (bp)
A1	Fw	GGAGGTTTTTATGCTTTAATATCTAAAGA	57	3,100
	Rv	CCCTCTGTTATTGTAGGTAGTACATTTA		
A2	Fw	TAAATGTACTACCTACAATAACAGAGGG	57	2,000
	Rv	CTTGTATATAAATCAGGTGCTATCAATA		
A3	*Fw*	TATTGATAGCACCTGATTTATATACAAG	47	3,100
	*Rv*	TTATCAAACATATATTTTAGCCATATATC		
B1	*Fw*	AGAAAATTTTATGAGTTTAGTTAATAGAAA	57	3,100
	*Rv*	CAGATAATGTAGGAAGTAAGTCTATAG		
B2	*Fw*	ATAGACTTACTTCCTACATTATCTGAA	47	2,000
	*Rv*	CATCTGTATAAATATTTGGTGAAATTAC		
B3	*Fw*	ATTTCACCAAATATTTATACAGATG	47	2,000
	*Rv*	ATTTAACATATTTTTATCTATTCA		

### Partial Sequencing of *tcd*A and *tcd*B Genes

Partial sequencing of 10 representative isolates, each of amplified *tcd*A and *tcd*B genes were performed to identify the nucleotide changes in them. The sequencing of fragments was carried out by Di-deoxy Sanger method. Comparison was made with known sequences of the *tcd*A and *tcd*B genes of reference strain *C. difficile* 630 (ribotype 012; toxinotype 0) using basic local alignment search tool (BLAST 2.2.29+) software accessed online from the National Center of Biotechnology Information (15). Nucleotide sequences alignment was done by multiple sequence comparison using log-expectation software and translated into amino acids by expert protein analysis system to detect the amino acid substitutions.

## Results

### Identification of Toxinotypes

**Figure 1:**
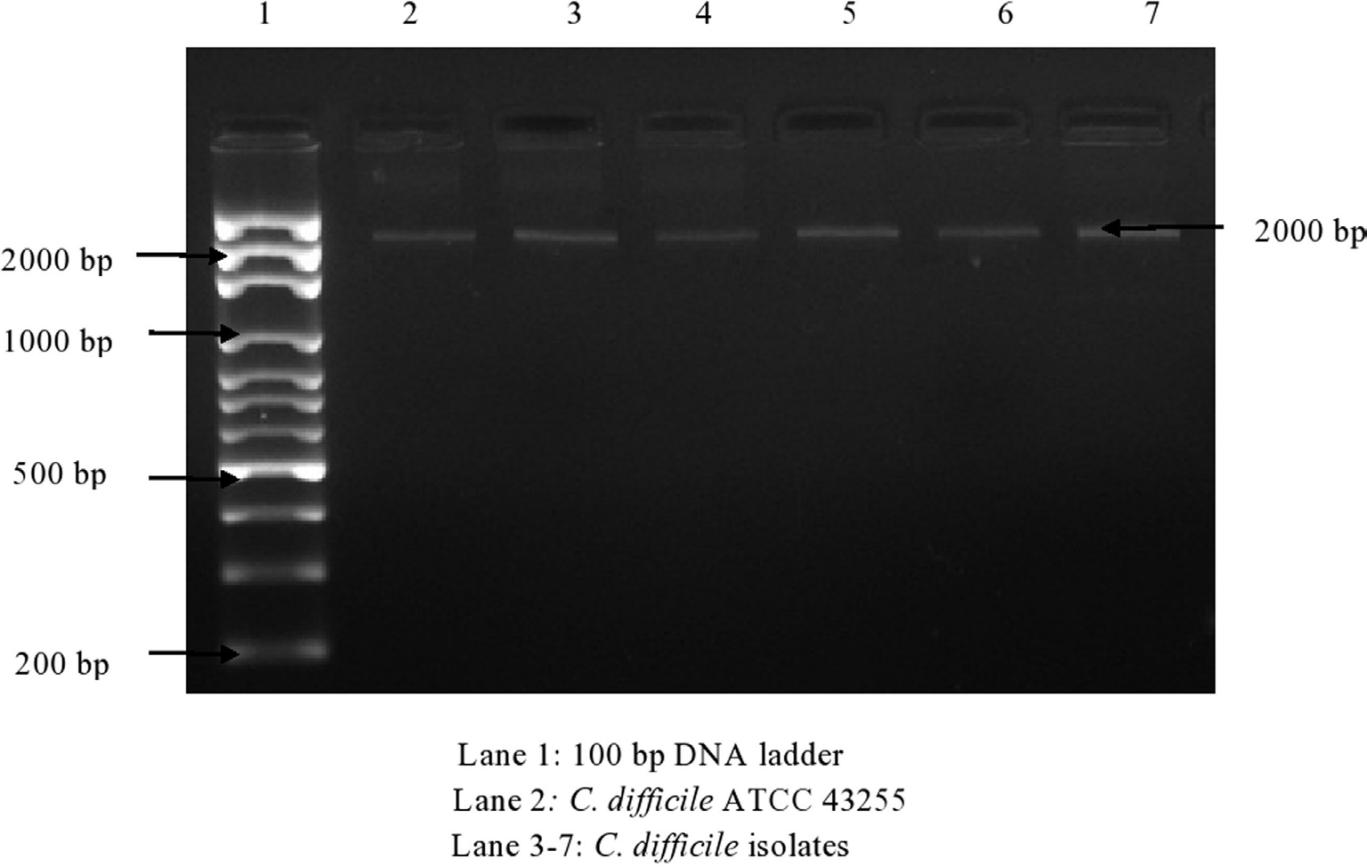
**PCR amplified fragment B3 of toxin B gene (2,000 bp)**.

**Figure 2:**
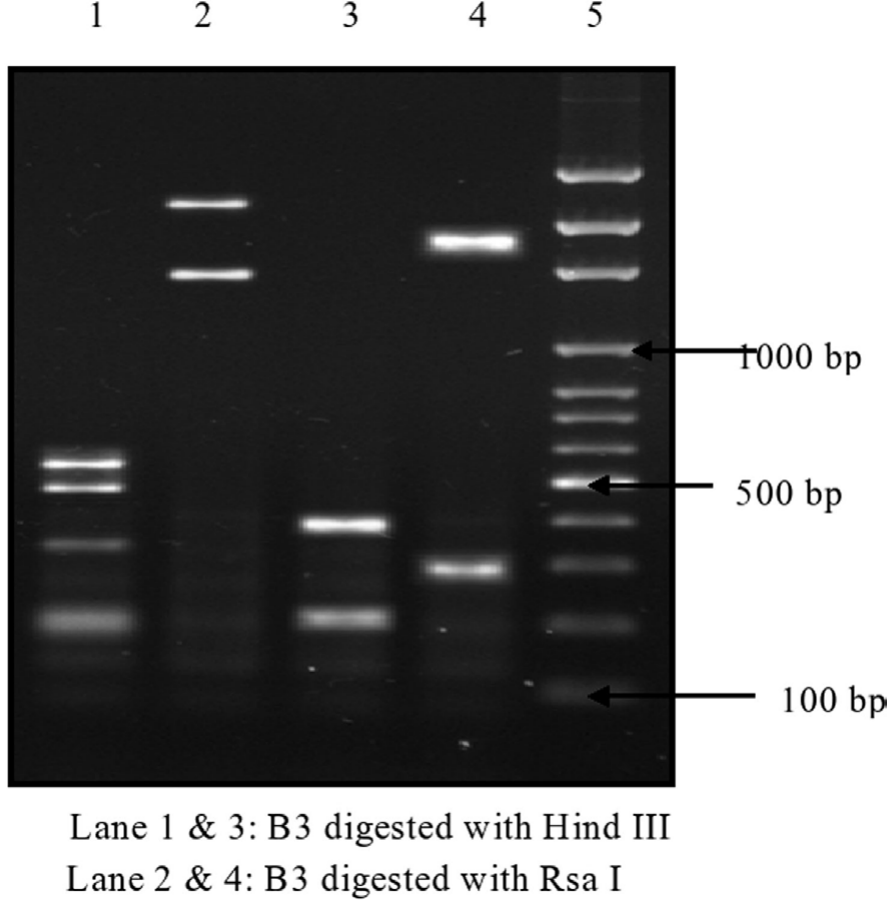
**Polymorphic restriction patterns of B3 fragment of *tcd* B gene by RFLP**.

Of 174 *C. difficile* isolates, 121 (69.5%) possessed either the *tcd*A or the *tcd*B gene or both. All of the toxigenic isolates were checked for the presence of fragments of toxins A1–A3 and B1–B3. Amplified toxin B3 fragment is shown in Figure 1. The fragments of toxins A and B after digestion with *Hin*dIII and *Rsa*I showed different polymorphic restriction patterns. Toxin B3 fragment digested with restriction enzymes is shown in Figure 2. Of the 121 toxigenic isolates, 76 (62.8%) belonged to toxinotype 0 and 45 (37.2%) to toxinotype VIII. Both *tcd*A and *tcd*B genes were present in 65 isolates of toxinotype 0. Four isolates of toxinotype 0 and nine of toxinotype VIII showed presence of only *tcd*A gene, whereas 36 isolates of toxinotype VIII had only *tcd*B gene. Relation of toxigenic genes to toxinotypes is depicted in Table 2.

**Table 2:** **Toxigenic genes in relation to toxinotypes**.

Toxigenic genes	Toxinotypes	
	0	VIII
*tcd*A^+^*tcd*B^+^	65	0
*tcd*A^+^*tcd*B^−^	4	9
*tcd*A^−^*tcd*B^+^	7	36
Total	76	45

### Sequences of Toxin A and B Genes

**Figure 3:**
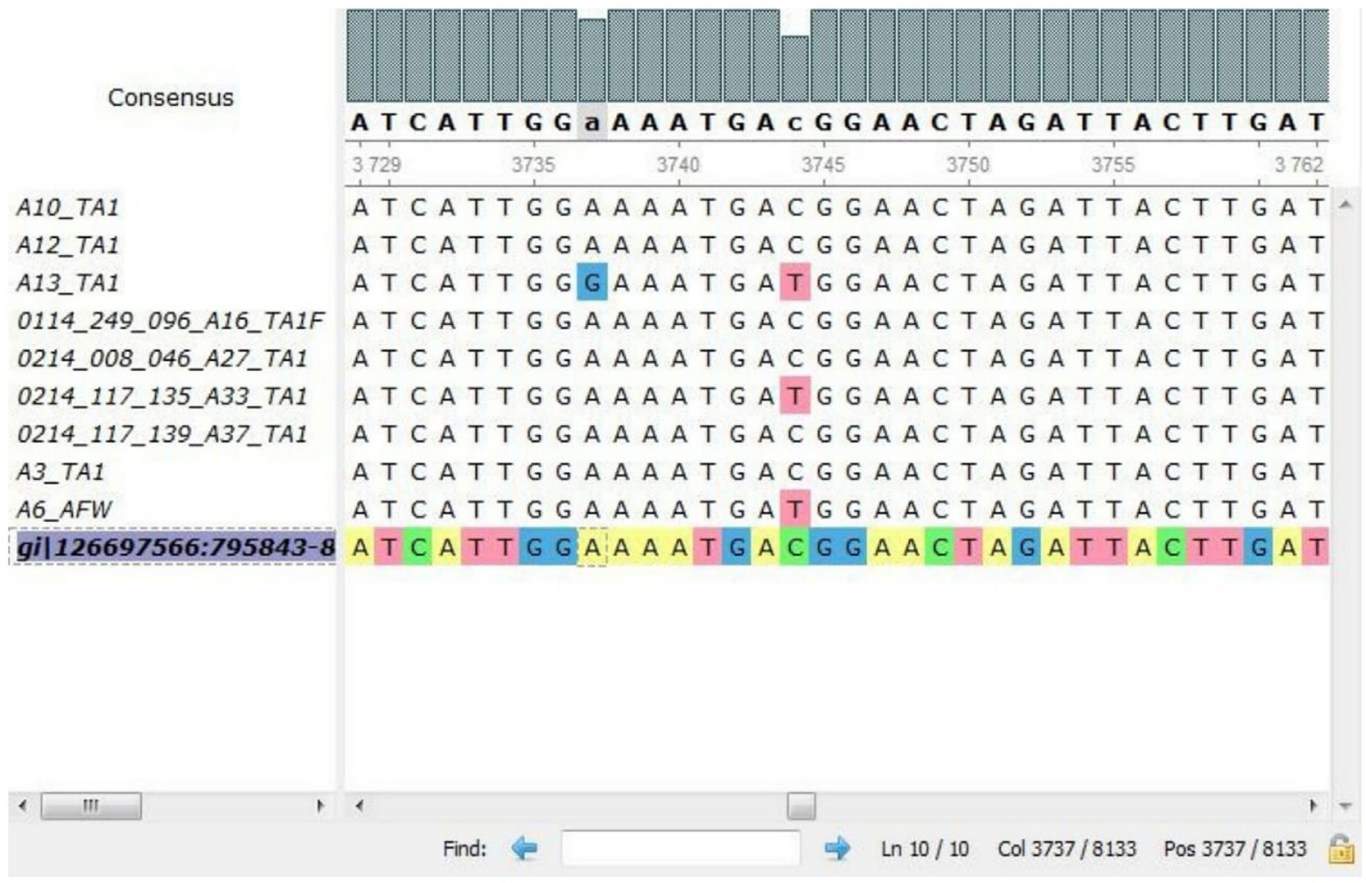
**Alignment of nucleotides for *tcd*A gene of representative isolates by multiple sequence comparison using log-expectation software**.

In all the representative isolates—10 each for toxin A and toxin B genes—the expected 624 and 591 bp bands of *tcd*A and *tcd*B, respectively, were found. The sequence of *tcd*A and *tcd*B genes of reference strain aligned with *tcd*A and *tcd*B genes of the isolates showed substitutions in toxin A sequences of five *C. difficile* isolates (Figure 3). In three isolates, cytosine was changed to thymine, and in another two isolates, adenine was replaced with guanine in *tcd*A gene (Table 3). In toxin B, there was no variation in the nucleotide sequences of any of the isolate. The annotated sequences of these isolates have been deposited with the National Center of Biotechnology, USA (Accession nos. KP182924-28).

**Table 3:** **Toxin A-positive isolates showing nucleotide substitutions at different positions**.

Isolate No.	Nucleotide substitution at different positions of *tcd*A gene				
A6	C 3338 T	C 3500 T		C 3774 T	T 3716 C
A9	C 3338 T	C 3500 T		C 3774 T	T 3716 C
A13			A 3667 G	C 3774 T	T 3716 C
A33		C 3500 T		C 3774 T	T 3716 C
A40			A 3667 G	C 3774 T	T 3716 C

## Discussion

The studies on CDI throughout Asia (9) and particularly India are limited due to the difficulty in culturing the pathogen as well as lack of funding. The current known burden of CDI in the local region is 17.5% (16). As reported earlier (13), toxigenic isolates were highly associated with cancers (77.3%), followed by surgery (75.0%) and gastrointestinal diseases (70.5%).

Due to the changes in the gene sequence of the toxin, the protein structure may also change leading to non-recognition by specific antibodies used in the diagnostic assays, and thus, cases of CDI may be missed. PCR amplification of toxigenic genes can help identify pathogenicity of *C. difficile* isolates because of its high sensitivity (17). As reported earlier (13), of the 121 toxigenic isolates, 68 (56.2%) possessed both the toxigenic genes (*tcd*A and *tcd*B), whereas the remaining 53 (43.8%) had one of the toxin genes. Only the toxin A (*tcd*A^+^*tcd*B^−^) gene was found in 13 (10.7%) and only the toxin B (*tcd*A^−^*tcd*B^+^) gene in 40 (33.1%) of the toxigenic isolates.

In the present study, 62.8% belonged to toxinotype 0 and 37.2% to toxinotype VIII. Both toxin genes *tcd*A and *tcd*B were present in 85.5% of toxinotype 0. Only *tcd*A gene was present in 5.3% of toxinotype 0 and 20.0% in toxinotype VIII, whereas *tcd*B gene was found in 5.3% of toxinotype 0 and 80.0% of toxinotype VIII. The toxinotypes observed in this study were similar to those observed from Europe, Asia, and elsewhere (9, 18–20). There are a few reports for exclusive expression of toxin B in Asian isolates. In Shanghai, 33.3% of the isolated strains were A^−^B^+^ strains, whereas in Stockholm, there was no A^−^B^+^ strain (21). In Korea, A^−^B^+^ variant was 25.7% (22), whereas in Iran, it was much lower than in Korea and Shanghai (23). However, there is no Indian study of *C. difficile* variants available as of date.

PCR ribotyping compares the patterns of PCR products of the 16S-23S rRNA intergenic spacer region (24). It has been established that toxinotyping and PCR ribotyping correlate well with each other (25). The 121 toxigenic *C. difficile* isolates belonged to ribotype 001 (36.8%), ribotype 017 (33.9%), and ribotype 106 (13.2%) as identified earlier (13), which is in accordance with other Asian studies where the most prevalent ribotypes were 001 and 017 (9). On comparison of ribotyping with the presence of toxin genes, our work complements and corroborates these studies.

Although the correlation of certain antibiotics with CDI is high, all antibiotics, inclusive of vancomycin and metronidazole, can cause CDI. Misuse of antibiotics can lead to the potential for spread of resistant organisms that can adversely impact the health of patients who are not even exposed to them. Detection of resistance pattern to certain antibiotics can be useful for epidemiological information and can benefit the clinical practitioners in treatment decision making. The drug resistance on these 174 isolates was reported earlier (12) where *C. difficile* showed a higher resistance toward clindamycin and ciprofloxacin but lower toward metronidazole. While comparing the antimicrobial resistance with the ribotypes, we found that the antimicrobial resistance rates were significantly different between each ribotype (data not shown). Comparison of the antibiotic resistogram profile of 121 toxigenic isolates with toxinotyping showed that three of the isolates resistant to metronidazole were of toxinotype 0. The most frequent toxinotypes resistant to ciprofloxacin were toxinotype 0 (54.2%), toxinotype VIII (26.5%), and toxinotype 0 (12.1%). None of the toxigenic isolates sensitive to vancomycin and metronidazole belonged to toxinotype 0. Forty-two percent of toxigenic isolates resistant to clindamycin belonged to toxinotype 0, 36.3% to toxinotype VIII, and 15.0% to toxinotype 0 (data not shown). Resistance to clindamycin was quite high among toxigenic isolates belonging to ribotype 001, toxinotype 0 (42.0%), and among ribotype 009 (14.5%) of the non-toxigenic isolates. Highest degree of antimicrobial resistance against ciprofloxacin was seen in ribotype 001 strain, while in ribotypes 017 and 106, lower resistance was found when compared with other ribotypes among toxigenic isolates. However, the limitation of this paper is that the antibiotic practice influencing the genetic variations in the region was not studied.

Several commercial kits available in earlier CDI diagnostic kits were designed to detect toxin A alone, and, therefore, A^−^B^+^ cases were getting missed. ELISA kits that detect only toxin A may miss out on toxins from isolates of A^−^B^+^ strains and thus result in wrong interpretation as the role of toxin B in causing CDI is equally important. Of 174 *C. difficile* isolates, 95 (54.6%) expressed toxins by ELISA (Paper under print). As of now, sequencing is the best technique to identify and to look for any variations in the genetic sequences and will be therefore helpful for clinicians to identify the genotype, consequently aiding in treatment decisions. In the present study, substitutions were found in *tcd*A sequences of five isolates but none in *tcd*B gene. Changes in the nucleotide sequence of *tcd*A gene suggest variation in the strains and highlight the usage of *C*. *difficile* diagnostic methods to detect both toxins A and B (26). Horizontal gene transfer could be responsible for the variability in the *C. difficile* DNA fragments amplified and sequenced (27). Molecular mechanism accountable for the lack of *tcd*A^−^ strains in 017 toxinotype could be due to sequence variation compared with toxigenic strains. Interestingly, NAP1/BI/027 isolates were not present in our study, and this absence corroborates with the less severe kind of CDI detected in the region. However, further study is required at other parts of the country.

## Conclusion

Outbreaks of *C. difficile* are a global phenomenon, and India has also risk of such outbreaks. Therefore, establishment of a global surveillance system is required for efficient control of CDI. Also, due to the widespread prevalence of ribotype 017 with only *tcd*B gene in Asia including India, the assay for identification of toxin B emerges to be more imperative than toxin A for diagnosis of CDI. Clinically, this study is useful in considering the ribotype and toxinotype trends in relation to antimicrobial resistance of *C. difficile* in India. Genetic variations in *C. difficile* isolates may alter the sensitivity and specificity of diagnostic assays. Moreover, the characterization of *C*. *difficile* is likely to facilitate and contribute to the development of an effective vaccine. Therefore, local data of the toxigenicity and genetic variations of *C. difficile* isolates can be very useful.

## Ethics Statement

Full name of the ethics committee: Institute Ethics Committee, Post Graduate Institute of Medical Education and Research, Chandigarh. Consent procedure: The patients whose fecal samples were collected were informed about the investigations to be conducted on their samples, and informed written consent was obtained. No vulnerable populations were involved.

## Author Contributions

CV conceived and designed the work, monitored the experiments, and supervised the final draft. RK provided the patients for clinical specimens and helped in drafting the paper. MS carried out the experiments, analyzed the data, performed literature search, and wrote the preliminary draft. SM approved the design, supervised the technical part of the experiments conducted, and approved the final draft.

## Conflict of Interest Statement

The authors declare that the research was conducted in the absence of any commercial or financial relationships that could be construed as a potential conflict of interest.
